# 5-[(*E*)-Meth­oxy(phen­yl)methyl­idene]-1,3,4-triphenyl-4,5-dihydro-1*H*-1,2,4-triazole

**DOI:** 10.1107/S1600536812045692

**Published:** 2012-11-10

**Authors:** Biplab Maji, Guillaume Berionni, Herbert Mayr, Peter Mayer

**Affiliations:** a Ludwig-Maximilians-Universität, Department Chemie und Biochemie, Butenandtstrasse 5-13, 81377 München, Germany

## Abstract

In the title compound, C_28_H_23_N_3_O, the 1,2,4-triazole ring deviates slightly from planarity adopting a ^N3^ *T*_C2_ conformation which is distorted towards an *E*_C2_ conformation. The plane around the ethyl­ene unit makes a dihedral angle of 17.32 (11)° with the mean plane [r.m.s. deviation = 0.036 (1) Å] of the 1,2,4-triazole fragment. The dihedral angles between the four phenyl rings and the 1,2,4-triazole ring are 31.01 (10), 49.01 (8), 78.55 (6) and 41.51 (9)°. In the crystal, mol­ecules are linked along [100] by weak C—H⋯O hydrogen bonds.

## Related literature

For chemical background, see: Arduengo *et al.* (1991 ▶); Enders *et al.* (2007 ▶); Biju *et al.* (2011 ▶); Breslow (1958 ▶). For puckering analysis, see: Cremer & Pople (1975 ▶). For a related structure, see: Nair *et al.* (2008 ▶).
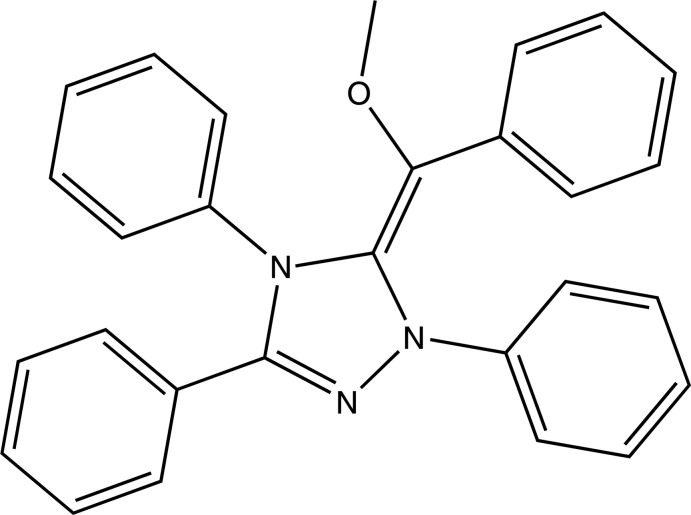


## Experimental

### 

#### Crystal data

C_28_H_23_N_3_O
*M* *_r_* = 417.49
Monoclinic, 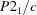

*a* = 5.8831 (2) Å
*b* = 10.5560 (2) Å
*c* = 35.0548 (8) Å
β = 93.749 (1)°
*V* = 2172.31 (10) Å^3^
*Z* = 4
Mo *K*α radiation
μ = 0.08 mm^−1^
*T* = 200 K
0.35 × 0.09 × 0.04 mm

#### Data collection

Nonius KappaCCD diffractometer
12170 measured reflections
3797 independent reflections
2601 reflections with *I* > 2σ(*I*)
*R*_int_ = 0.058

#### Refinement

*R*[*F* ^2^ > 2σ(*F* ^2^)] = 0.045
*wR*(*F* ^2^) = 0.116
*S* = 1.02
3797 reflections
290 parameters
H-atom parameters constrained
Δρ_max_ = 0.14 e Å^−3^
Δρ_min_ = −0.17 e Å^−3^

### 

Data collection: *COLLECT* (Nonius, 1998) ▶; cell refinement: *SCALEPACK* (Otwinowski & Minor, 1997 ▶); data reduction: *DENZO* (Otwinowski & Minor, 1997 ▶) and *SCALEPACK*; program(s) used to solve structure: *SIR97* (Altomare *et al.*, 1999 ▶); program(s) used to refine structure: *SHELXL97* (Sheldrick, 2008 ▶); molecular graphics: *ORTEP-3* (Farrugia, 1997 ▶); software used to prepare material for publication: *PLATON* (Spek, 2009 ▶).

## Supplementary Material

Crystal structure: contains datablock(s) I, global. DOI: 10.1107/S1600536812045692/lx2259sup1.cif

Structure factors: contains datablock(s) I. DOI: 10.1107/S1600536812045692/lx2259Isup2.hkl

Supplementary material file. DOI: 10.1107/S1600536812045692/lx2259Isup3.cml

Additional supplementary materials:  crystallographic information; 3D view; checkCIF report

## Figures and Tables

**Table 1:** Hydrogen-bond geometry (Å, °)

*D*—H⋯*A*	*D*—H	H⋯*A*	*D*⋯*A*	*D*—H⋯*A*
C20—H20⋯O1^i^	0.95	2.43	3.162 (3)	134

Symmetry code: (i) 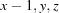.

**Fig. 1.**
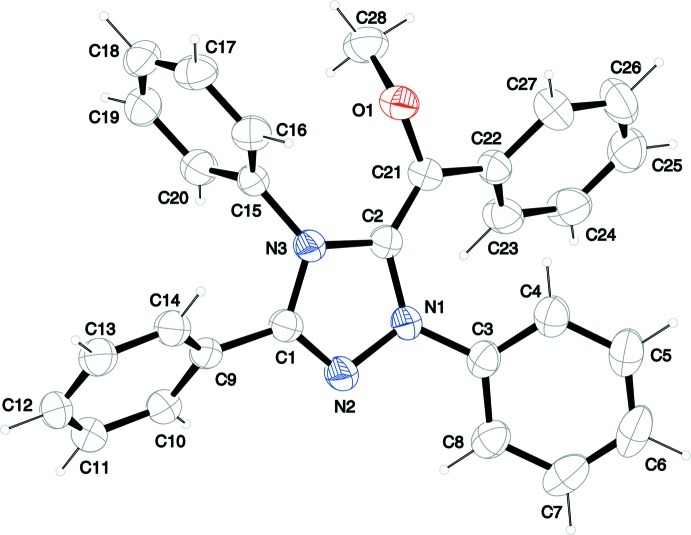
The molecular structure of the title compound with the atom numbering scheme. Displacement ellipsoids are drawn at the 50% probability level. H atoms are presented as small spheres of arbitrary radius.

## supplementary crystallographic information

### Comment

Umpolung reactions of aldehydes catalyzed by N-heterocyclic carbenes [Arduengo *et al.* (1991)] belong to the most important organocatalytic CC bond-forming reactions [Enders *et al.* (2007), Biju *et al.* (2011)]. An acyl anion equivalent, the so called Breslow intermediate [Breslow (1958)] was proposed to be the key intermediate of these transformations.

To understand the structure of these intermediates, we report herein the crystal structure of the title compound.

In the title molecule (Fig. 1), the 1,2,4-triazole fragment is nearly planar, with a mean deviation of 0.036 (1) Å from the least-squares plane defined by the five constituent atoms.

The asymmetric unit contains one molecule of the title compound which is shown in Figure 1.

Puckering analysis [Cremer *et al.* (1975)] reveals that the 4,5-dihydro-1*H*-1,2,4-triazole ring adopts a ^N3^*T*_C2_ conformation which is distorted towards a *E*_C2_ conformation. In a related structure [Nair *et al.* (2008)] of a compound which is different from the title compound only through the substituent connected by a double bond with the 4,5-dihydro-1*H*-1,2,4-triazole ring, the latter ring is planar and not puckered. The methoxy(phenyl)methylene group is bound to the 4,5-dihydro-1*H*-1,2,4-triazole ring in a distance of 1.358 (3) Å which indicates a double bond. However, the plane of the heterocycle and the plane around the methylene atom are not coplanar but enclose a dihedral angle of 17.32 (11)°. In the related structure, the corresponding dihedral angle is 42.5 (4)°. The plane of the phenyl ring bound to the methylene atom is not coplanar with the plane around the methylene atom as well (dihedral angle 34.14 (10)°). The dihedral angles between the four phenyl rings and the mean plane of the 1,2,4-triazole ring are 31.01 (10)° [the C3-C8 phenyl ring], 49.01 (8)° [the C9-C14 phenyl ring], 78.55 (6)° [the C15-C20 phenyl ring] and 41.51 (9)° [the C22-C27 phenyl ring]. In the crystal packing, molecules are connected by weak C–H···O hydrogen bonds (Table 1).

### Experimental

To an oven dried Schlenk-flask charged with NaH (48 mg, 2.0 mmol), *t*BuOK (11 mg, 98 mol), and 5-(methoxy(phenyl)methyl)-1,3,4-triphenyl-4*H*-1,2,4-triazolium chloride (454 mg, 1.00 mmol) was added dry THF (15 ml) under nitrogen and the reaction mixture was allowed to stir for 4 h in the dark. The solvent was then removed under vacuum, and the residue was suspended in dry toluene (20 ml) and filtered through a celite pad under nitrogen. Then the solvent was evaporated to give 296 mg (0.709 mmol, 71%) of the title compound as 10:1 mixture of *E*:*Z* isomers. Crystals suitable for X-ray crystallography were grown by cooling down a saturated acetonitrile solution at -30 °C under argon for 48 h.

### Refinement

C-bound H atoms were positioned geometrically (C–H = 0.98 Å for aliphatic, 0.95 Å for aromatic H) and treated as riding on their parent atoms [*U*_iso_(H) = 1.2*U*_eq_(C, aromatic), *U*_iso_(H) = 1.5*U*_eq_(C, aliphatic)]. The methyl group was allowed to rotate along the C-O bond to best fit the experimental electron density.

### Crystal data

**Table:**

C_28_H_23_N_3_O	*F*(000) = 880
*M**_r_* = 417.49	*D*_x_ = 1.277 Mg m^−^^3^
Monoclinic, *P*2_1_/*c*	Mo *K*α radiation, λ = 0.71073 Å
Hall symbol: -P 2ybc	Cell parameters from 6637 reflections
*a* = 5.8831 (2) Å	θ = 3.1–25.4°
*b* = 10.5560 (2) Å	µ = 0.08 mm^−^^1^
*c* = 35.0548 (8) Å	*T* = 200 K
β = 93.749 (1)°	Rod, yellow
*V* = 2172.31 (10) Å^3^	0.35 × 0.09 × 0.04 mm
*Z* = 4	

### Data collection

**Table:**

Nonius KappaCCD diffractometer	2601 reflections with *I* > 2σ(*I*)
Radiation source: rotating anode	*R*_int_ = 0.058
MONTEL, graded multilayered X-ray optics monochromator	θ_max_ = 25.0°, θ_min_ = 3.5°
Detector resolution: 9 pixels mm^-1^	*h* = −6→6
CCD; rotation images scans	*k* = −12→12
12170 measured reflections	*l* = −41→41
3797 independent reflections	

### Refinement

**Table:**

Refinement on *F*^2^	Primary atom site location: structure-invariant direct methods
Least-squares matrix: full	Secondary atom site location: difference Fourier map
*R*[*F*^2^ > 2σ(*F*^2^)] = 0.045	Hydrogen site location: inferred from neighbouring sites
*wR*(*F*^2^) = 0.116	H-atom parameters constrained
*S* = 1.02	*w* = 1/[σ^2^(*F*_o_^2^) + (0.0489*P*)^2^ + 0.5794*P*] where *P* = (*F*_o_^2^ + 2*F*_c_^2^)/3
3797 reflections	(Δ/σ)_max_ < 0.001
290 parameters	Δρ_max_ = 0.14 e Å^−^^3^
0 restraints	Δρ_min_ = −0.17 e Å^−^^3^

### Special details

**Table:**

Geometry. All e.s.d.'s (except the e.s.d. in the dihedral angle between two l.s. planes) are estimated using the full covariance matrix. The cell e.s.d.'s are taken into account individually in the estimation of e.s.d.'s in distances, angles and torsion angles; correlations between e.s.d.'s in cell parameters are only used when they are defined by crystal symmetry. An approximate (isotropic) treatment of cell e.s.d.'s is used for estimating e.s.d.'s involving l.s. planes.
Refinement. Refinement of *F*^2^ against ALL reflections. The weighted *R*-factor *wR* and goodness of fit *S* are based on *F*^2^, conventional *R*-factors *R* are based on *F*, with *F* set to zero for negative *F*^2^. The threshold expression of *F*^2^ > 2*σ*(*F*^2^) is used only for calculating *R*-factors(gt) *etc*. and is not relevant to the choice of reflections for refinement. *R*-factors based on *F*^2^ are statistically about twice as large as those based on *F*, and *R*-factors based on all data will be even larger.

### Fractional atomic coordinates and isotropic or equivalent isotropic displacement parameters (Å^2^)

**Table:**

	*x*	*y*	*z*	*U*_iso_*/*U*_eq_	
O1	1.1775 (2)	1.09309 (12)	0.07479 (4)	0.0400 (4)	
N1	1.0129 (3)	0.87548 (14)	0.15014 (4)	0.0372 (4)	
N2	0.8760 (3)	0.91293 (15)	0.17968 (5)	0.0397 (4)	
N3	0.9369 (3)	1.07815 (14)	0.14195 (4)	0.0334 (4)	
C1	0.8328 (3)	1.03112 (18)	0.17323 (5)	0.0343 (5)	
C2	1.0374 (3)	0.97436 (17)	0.12411 (5)	0.0328 (5)	
C3	1.1547 (3)	0.76856 (18)	0.15771 (5)	0.0362 (5)	
C4	1.3673 (4)	0.7594 (2)	0.14287 (6)	0.0428 (5)	
H4	1.4222	0.8266	0.1280	0.051*	
C5	1.4982 (4)	0.6526 (2)	0.14986 (6)	0.0513 (6)	
H5	1.6422	0.6459	0.1392	0.062*	
C6	1.4236 (4)	0.5561 (2)	0.17190 (7)	0.0579 (7)	
H6	1.5132	0.4819	0.1761	0.070*	
C7	1.2164 (4)	0.5677 (2)	0.18806 (6)	0.0539 (6)	
H7	1.1665	0.5025	0.2042	0.065*	
C8	1.0811 (4)	0.67325 (18)	0.18098 (6)	0.0429 (5)	
H8	0.9384	0.6802	0.1920	0.051*	
C9	0.6887 (3)	1.10815 (17)	0.19698 (5)	0.0347 (5)	
C10	0.4797 (4)	1.06009 (19)	0.20649 (6)	0.0397 (5)	
H10	0.4299	0.9795	0.1972	0.048*	
C11	0.3445 (4)	1.1301 (2)	0.22955 (6)	0.0451 (5)	
H11	0.2019	1.0971	0.2361	0.054*	
C12	0.4148 (4)	1.2471 (2)	0.24312 (6)	0.0481 (6)	
H12	0.3214	1.2947	0.2589	0.058*	
C13	0.6216 (4)	1.2945 (2)	0.23355 (6)	0.0477 (6)	
H13	0.6703	1.3751	0.2429	0.057*	
C14	0.7589 (4)	1.22654 (18)	0.21059 (6)	0.0415 (5)	
H14	0.9009	1.2604	0.2041	0.050*	
C15	0.8681 (3)	1.19569 (17)	0.12357 (5)	0.0325 (5)	
C16	1.0135 (4)	1.29802 (18)	0.12531 (6)	0.0399 (5)	
H16	1.1605	1.2911	0.1381	0.048*	
C17	0.9442 (4)	1.41053 (19)	0.10842 (6)	0.0464 (6)	
H17	1.0438	1.4815	0.1094	0.056*	
C18	0.7300 (4)	1.4201 (2)	0.09014 (6)	0.0497 (6)	
H18	0.6830	1.4976	0.0783	0.060*	
C19	0.5839 (4)	1.3179 (2)	0.08887 (6)	0.0493 (6)	
H19	0.4358	1.3253	0.0765	0.059*	
C20	0.6534 (4)	1.20490 (19)	0.10556 (6)	0.0414 (5)	
H20	0.5540	1.1339	0.1047	0.050*	
C21	1.1253 (3)	0.97381 (18)	0.08928 (5)	0.0355 (5)	
C22	1.1836 (3)	0.86455 (18)	0.06625 (5)	0.0368 (5)	
C23	1.0535 (4)	0.75417 (19)	0.06459 (6)	0.0450 (5)	
H23	0.9233	0.7483	0.0792	0.054*	
C24	1.1113 (4)	0.6529 (2)	0.04203 (6)	0.0533 (6)	
H24	1.0203	0.5785	0.0412	0.064*	
C25	1.2999 (5)	0.6594 (2)	0.02071 (7)	0.0588 (7)	
H25	1.3400	0.5897	0.0054	0.071*	
C26	1.4289 (4)	0.7680 (2)	0.02195 (7)	0.0594 (7)	
H26	1.5592	0.7731	0.0074	0.071*	
C27	1.3718 (4)	0.8695 (2)	0.04401 (6)	0.0499 (6)	
H27	1.4619	0.9442	0.0441	0.060*	
C28	1.0477 (4)	1.1273 (2)	0.04035 (6)	0.0510 (6)	
H28A	1.1075	1.0825	0.0187	0.077*	
H28B	1.0590	1.2189	0.0363	0.077*	
H28C	0.8878	1.1040	0.0425	0.077*	

### Atomic displacement parameters (Å^2^)

**Table:**

	*U*^11^	*U*^22^	*U*^33^	*U*^12^	*U*^13^	*U*^23^
O1	0.0444 (9)	0.0394 (8)	0.0369 (8)	−0.0049 (7)	0.0075 (6)	0.0010 (6)
N1	0.0436 (10)	0.0337 (9)	0.0356 (9)	0.0083 (8)	0.0126 (8)	0.0004 (7)
N2	0.0493 (11)	0.0336 (10)	0.0378 (10)	0.0050 (8)	0.0148 (8)	−0.0009 (7)
N3	0.0384 (10)	0.0302 (8)	0.0323 (9)	0.0020 (7)	0.0084 (7)	0.0001 (7)
C1	0.0369 (12)	0.0326 (11)	0.0340 (11)	−0.0005 (9)	0.0064 (9)	0.0008 (9)
C2	0.0330 (11)	0.0319 (10)	0.0336 (11)	0.0006 (9)	0.0030 (9)	0.0000 (9)
C3	0.0426 (13)	0.0344 (11)	0.0312 (11)	0.0057 (10)	−0.0011 (9)	−0.0055 (9)
C4	0.0450 (14)	0.0458 (13)	0.0370 (12)	0.0076 (11)	−0.0017 (10)	−0.0063 (10)
C5	0.0514 (15)	0.0560 (15)	0.0451 (13)	0.0192 (12)	−0.0066 (11)	−0.0098 (11)
C6	0.0674 (18)	0.0483 (15)	0.0555 (15)	0.0257 (13)	−0.0155 (13)	−0.0093 (12)
C7	0.0787 (19)	0.0355 (12)	0.0454 (14)	0.0039 (12)	−0.0113 (13)	0.0016 (10)
C8	0.0542 (14)	0.0358 (11)	0.0381 (12)	0.0029 (10)	−0.0012 (10)	−0.0015 (9)
C9	0.0426 (13)	0.0336 (11)	0.0284 (10)	0.0058 (9)	0.0065 (9)	0.0029 (8)
C10	0.0449 (13)	0.0359 (11)	0.0390 (12)	0.0040 (10)	0.0080 (10)	0.0032 (9)
C11	0.0481 (14)	0.0484 (13)	0.0398 (12)	0.0090 (11)	0.0116 (10)	0.0072 (10)
C12	0.0619 (16)	0.0417 (13)	0.0427 (12)	0.0172 (12)	0.0189 (11)	0.0016 (10)
C13	0.0670 (16)	0.0351 (12)	0.0419 (13)	0.0070 (11)	0.0117 (11)	−0.0023 (9)
C14	0.0497 (13)	0.0352 (11)	0.0404 (12)	0.0020 (10)	0.0094 (10)	−0.0003 (9)
C15	0.0382 (12)	0.0308 (10)	0.0294 (11)	0.0030 (9)	0.0080 (9)	−0.0002 (8)
C16	0.0429 (13)	0.0392 (12)	0.0375 (12)	−0.0044 (10)	0.0018 (10)	0.0011 (9)
C17	0.0629 (16)	0.0356 (12)	0.0409 (12)	−0.0064 (11)	0.0061 (11)	0.0013 (10)
C18	0.0699 (17)	0.0383 (12)	0.0420 (13)	0.0154 (12)	0.0118 (12)	0.0055 (10)
C19	0.0459 (14)	0.0576 (15)	0.0445 (14)	0.0124 (12)	0.0032 (10)	0.0075 (11)
C20	0.0387 (13)	0.0453 (12)	0.0405 (12)	0.0000 (10)	0.0044 (10)	0.0036 (10)
C21	0.0358 (12)	0.0368 (11)	0.0342 (11)	−0.0008 (9)	0.0061 (9)	0.0026 (9)
C22	0.0378 (12)	0.0417 (12)	0.0311 (11)	0.0042 (10)	0.0040 (9)	−0.0001 (9)
C23	0.0575 (15)	0.0437 (12)	0.0345 (12)	−0.0014 (11)	0.0092 (10)	−0.0017 (10)
C24	0.0801 (18)	0.0407 (13)	0.0395 (13)	0.0006 (12)	0.0069 (13)	−0.0013 (10)
C25	0.0808 (19)	0.0521 (15)	0.0442 (14)	0.0205 (14)	0.0095 (13)	−0.0076 (11)
C26	0.0567 (16)	0.0711 (17)	0.0526 (15)	0.0123 (14)	0.0199 (12)	−0.0093 (13)
C27	0.0448 (14)	0.0585 (14)	0.0475 (13)	−0.0009 (11)	0.0119 (11)	−0.0046 (11)
C28	0.0662 (16)	0.0509 (13)	0.0359 (12)	0.0018 (12)	0.0034 (11)	0.0062 (10)

### Geometric parameters (Å, º)

**Table:**

O1—C21	1.399 (2)	C13—C14	1.378 (3)
O1—C28	1.432 (2)	C13—H13	0.9500
N1—C2	1.400 (2)	C14—H14	0.9500
N1—N2	1.410 (2)	C15—C16	1.377 (3)
N1—C3	1.418 (2)	C15—C20	1.378 (3)
N2—C1	1.290 (2)	C16—C17	1.377 (3)
N3—C1	1.383 (2)	C16—H16	0.9500
N3—C2	1.411 (2)	C17—C18	1.380 (3)
N3—C15	1.444 (2)	C17—H17	0.9500
C1—C9	1.471 (3)	C18—C19	1.378 (3)
C2—C21	1.357 (3)	C18—H18	0.9500
C3—C8	1.383 (3)	C19—C20	1.379 (3)
C3—C4	1.389 (3)	C19—H19	0.9500
C4—C5	1.379 (3)	C20—H20	0.9500
C4—H4	0.9500	C21—C22	1.461 (3)
C5—C6	1.368 (3)	C22—C23	1.393 (3)
C5—H5	0.9500	C22—C27	1.396 (3)
C6—C7	1.383 (3)	C23—C24	1.385 (3)
C6—H6	0.9500	C23—H23	0.9500
C7—C8	1.382 (3)	C24—C25	1.379 (3)
C7—H7	0.9500	C24—H24	0.9500
C8—H8	0.9500	C25—C26	1.374 (3)
C9—C14	1.391 (3)	C25—H25	0.9500
C9—C10	1.391 (3)	C26—C27	1.375 (3)
C10—C11	1.384 (3)	C26—H26	0.9500
C10—H10	0.9500	C27—H27	0.9500
C11—C12	1.377 (3)	C28—H28A	0.9800
C11—H11	0.9500	C28—H28B	0.9800
C12—C13	1.377 (3)	C28—H28C	0.9800
C12—H12	0.9500		
			
C21—O1—C28	114.57 (15)	C13—C14—H14	120.1
C2—N1—N2	110.97 (14)	C9—C14—H14	120.2
C2—N1—C3	129.13 (16)	C16—C15—C20	120.87 (18)
N2—N1—C3	116.30 (15)	C16—C15—N3	119.97 (17)
C1—N2—N1	104.95 (15)	C20—C15—N3	119.13 (17)
C1—N3—C2	107.04 (15)	C15—C16—C17	119.50 (19)
C1—N3—C15	122.47 (15)	C15—C16—H16	120.3
C2—N3—C15	125.66 (15)	C17—C16—H16	120.3
N2—C1—N3	113.19 (17)	C16—C17—C18	119.9 (2)
N2—C1—C9	123.34 (17)	C16—C17—H17	120.1
N3—C1—C9	123.48 (16)	C18—C17—H17	120.1
C21—C2—N1	130.15 (17)	C19—C18—C17	120.4 (2)
C21—C2—N3	126.72 (17)	C19—C18—H18	119.8
N1—C2—N3	103.07 (15)	C17—C18—H18	119.8
C8—C3—C4	119.67 (19)	C18—C19—C20	119.8 (2)
C8—C3—N1	119.11 (18)	C18—C19—H19	120.1
C4—C3—N1	121.21 (18)	C20—C19—H19	120.1
C5—C4—C3	119.8 (2)	C15—C20—C19	119.5 (2)
C5—C4—H4	120.1	C15—C20—H20	120.2
C3—C4—H4	120.1	C19—C20—H20	120.2
C6—C5—C4	120.9 (2)	C2—C21—O1	115.37 (16)
C6—C5—H5	119.6	C2—C21—C22	128.13 (17)
C4—C5—H5	119.6	O1—C21—C22	116.40 (16)
C5—C6—C7	119.3 (2)	C23—C22—C27	117.48 (19)
C5—C6—H6	120.3	C23—C22—C21	122.35 (18)
C7—C6—H6	120.3	C27—C22—C21	120.15 (18)
C8—C7—C6	120.7 (2)	C24—C23—C22	121.0 (2)
C8—C7—H7	119.6	C24—C23—H23	119.5
C6—C7—H7	119.6	C22—C23—H23	119.5
C7—C8—C3	119.6 (2)	C25—C24—C23	120.5 (2)
C7—C8—H8	120.2	C25—C24—H24	119.8
C3—C8—H8	120.2	C23—C24—H24	119.8
C14—C9—C10	119.54 (18)	C26—C25—C24	119.1 (2)
C14—C9—C1	121.43 (19)	C26—C25—H25	120.4
C10—C9—C1	119.02 (17)	C24—C25—H25	120.4
C11—C10—C9	119.74 (19)	C25—C26—C27	120.8 (2)
C11—C10—H10	120.1	C25—C26—H26	119.6
C9—C10—H10	120.1	C27—C26—H26	119.6
C12—C11—C10	120.6 (2)	C26—C27—C22	121.1 (2)
C12—C11—H11	119.7	C26—C27—H27	119.4
C10—C11—H11	119.7	C22—C27—H27	119.4
C13—C12—C11	119.5 (2)	O1—C28—H28A	109.5
C13—C12—H12	120.3	O1—C28—H28B	109.5
C11—C12—H12	120.3	H28A—C28—H28B	109.5
C12—C13—C14	120.9 (2)	O1—C28—H28C	109.5
C12—C13—H13	119.5	H28A—C28—H28C	109.5
C14—C13—H13	119.5	H28B—C28—H28C	109.5
C13—C14—C9	119.7 (2)		
			
C2—N1—N2—C1	−4.0 (2)	C10—C11—C12—C13	0.0 (3)
C3—N1—N2—C1	156.58 (17)	C11—C12—C13—C14	0.1 (3)
N1—N2—C1—N3	−1.9 (2)	C12—C13—C14—C9	−0.3 (3)
N1—N2—C1—C9	178.51 (17)	C10—C9—C14—C13	0.5 (3)
C2—N3—C1—N2	7.0 (2)	C1—C9—C14—C13	−178.36 (18)
C15—N3—C1—N2	163.63 (17)	C1—N3—C15—C16	112.9 (2)
C2—N3—C1—C9	−173.41 (17)	C2—N3—C15—C16	−95.0 (2)
C15—N3—C1—C9	−16.8 (3)	C1—N3—C15—C20	−64.9 (2)
N2—N1—C2—C21	−169.4 (2)	C2—N3—C15—C20	87.3 (2)
C3—N1—C2—C21	33.2 (3)	C20—C15—C16—C17	−0.8 (3)
N2—N1—C2—N3	7.94 (19)	N3—C15—C16—C17	−178.53 (18)
C3—N1—C2—N3	−149.47 (18)	C15—C16—C17—C18	0.4 (3)
C1—N3—C2—C21	168.75 (19)	C16—C17—C18—C19	0.5 (3)
C15—N3—C2—C21	13.1 (3)	C17—C18—C19—C20	−0.8 (3)
C1—N3—C2—N1	−8.70 (19)	C16—C15—C20—C19	0.4 (3)
C15—N3—C2—N1	−164.34 (16)	N3—C15—C20—C19	178.17 (18)
C2—N1—C3—C8	−171.14 (18)	C18—C19—C20—C15	0.4 (3)
N2—N1—C3—C8	32.4 (2)	N1—C2—C21—O1	−164.52 (18)
C2—N1—C3—C4	10.3 (3)	N3—C2—C21—O1	18.7 (3)
N2—N1—C3—C4	−146.16 (18)	N1—C2—C21—C22	11.7 (3)
C8—C3—C4—C5	3.3 (3)	N3—C2—C21—C22	−165.01 (19)
N1—C3—C4—C5	−178.13 (17)	C28—O1—C21—C2	−116.42 (19)
C3—C4—C5—C6	−1.3 (3)	C28—O1—C21—C22	66.9 (2)
C4—C5—C6—C7	−1.5 (3)	C2—C21—C22—C23	37.1 (3)
C5—C6—C7—C8	2.4 (3)	O1—C21—C22—C23	−146.70 (18)
C6—C7—C8—C3	−0.5 (3)	C2—C21—C22—C27	−144.7 (2)
C4—C3—C8—C7	−2.4 (3)	O1—C21—C22—C27	31.5 (3)
N1—C3—C8—C7	178.99 (17)	C27—C22—C23—C24	0.8 (3)
N2—C1—C9—C14	131.4 (2)	C21—C22—C23—C24	179.02 (19)
N3—C1—C9—C14	−48.1 (3)	C22—C23—C24—C25	0.1 (3)
N2—C1—C9—C10	−47.4 (3)	C23—C24—C25—C26	−0.4 (3)
N3—C1—C9—C10	133.1 (2)	C24—C25—C26—C27	−0.1 (4)
C14—C9—C10—C11	−0.4 (3)	C25—C26—C27—C22	1.0 (4)
C1—C9—C10—C11	178.48 (18)	C23—C22—C27—C26	−1.3 (3)
C9—C10—C11—C12	0.1 (3)	C21—C22—C27—C26	−179.6 (2)

### Hydrogen-bond geometry (Å, º)

**Table:**

*D*—H···*A*	*D*—H	H···*A*	*D*···*A*	*D*—H···*A*
C20—H20···O1^i^	0.95	2.43	3.162 (3)	134

Symmetry code: (i) *x*−1, *y*, *z*.
